# Structural basis of nSH2 regulation and lipid binding in PI3Kα

**DOI:** 10.18632/oncotarget.2263

**Published:** 2014-07-25

**Authors:** Michelle S. Miller, Oleg Schmidt-Kittler, David M. Bolduc, Evan T. Brower, Daniele Chaves-Moreira, Marc Allaire, Kenneth W. Kinzler, Ian G. Jennings, Philip E. Thompson, Philip A. Cole, L. Mario Amzel, Bert Vogelstein, Sandra B. Gabelli

**Affiliations:** ^1^ Medicinal Chemistry, Monash Institute of Pharmaceutical Sciences, Parkville, Victoria, Australia; ^2^ Ludwig Center for Cancer Genetics and Therapeutics and Howard Hughes Medical Institutions, Johns Hopkins University School of Medicine, Baltimore, Maryland, USA; ^3^ Department of Pharmacology and Molecular Sciences, Johns Hopkins University School of Medicine, Baltimore, Maryland, USA; ^4^ Department of Biophysics and Biophysical Chemistry, Johns Hopkins University School of Medicine, Baltimore, Maryland, USA; ^5^ Department of Medicine, Johns Hopkins University School of Medicine, Baltimore, Maryland, USA; ^6^ Department of Oncology, Johns Hopkins University School of Medicine, Baltimore, Maryland, USA; ^7^ Photon Sciences, Brookhaven National Laboratory, Upton, New York, USA; ^8^ Present Address: Department of Oncology, Johns Hopkins University School of Medicine, Baltimore Maryland, USA; ^9^ Present Address: Sanofi, Cambridge, Massachusetts; ^10^ Present Address: Center for Neurologic Diseases, Brigham and Women's Hospital and Harvard Medical School, Boston, Massachusetts; ^11^ Present Address: Paragon Bioservices, Baltimore, Maryland; ^12^ Present Address: Berkeley Center for Structural Biology, Physical Biosciences Division, Lawrence Berkeley National Laboratory, Berkeley, California

**Keywords:** PIK3R1, p85, PIK3CA, PI3K, PIP_2_, PIP_3_

## Abstract

We report two crystal structures of the wild-type phosphatidylinositol 3-kinase α (PI3Kα) heterodimer refined to 2.9 Å and 3.4 Å resolution: the first as the free enzyme, the second in complex with the lipid substrate, diC4-PIP_2_, respectively. The first structure shows key interactions of the N-terminal SH2 domain (nSH2) and iSH2 with the activation loop that suggest a mechanism by which the enzyme is inhibited in its basal state. In the second structure, the lipid substrate binds in a positively charged pocket adjacent to the ATP-binding site, bordered by the P-loop, the activation loop and the iSH2 domain. An additional lipid-binding site was identified at the interface of the ABD, iSH2 and kinase domains. The ability of PI3Kα to bind an additional PIP_2_ molecule was confirmed in vitro by fluorescence quenching experiments. The crystal structures reveal key differences in the way the nSH2 domain interacts with wild-type p110α and with the oncogenic mutant p110αH1047R. Increased buried surface area and two unique salt-bridges observed only in the wild-type structure suggest tighter inhibition in the wild-type PI3Kα than in the oncogenic mutant. These differences may be partially responsible for the increased basal lipid kinase activity and increased membrane binding of the oncogenic mutant.

## INTRODUCTION

**Fig 1:**
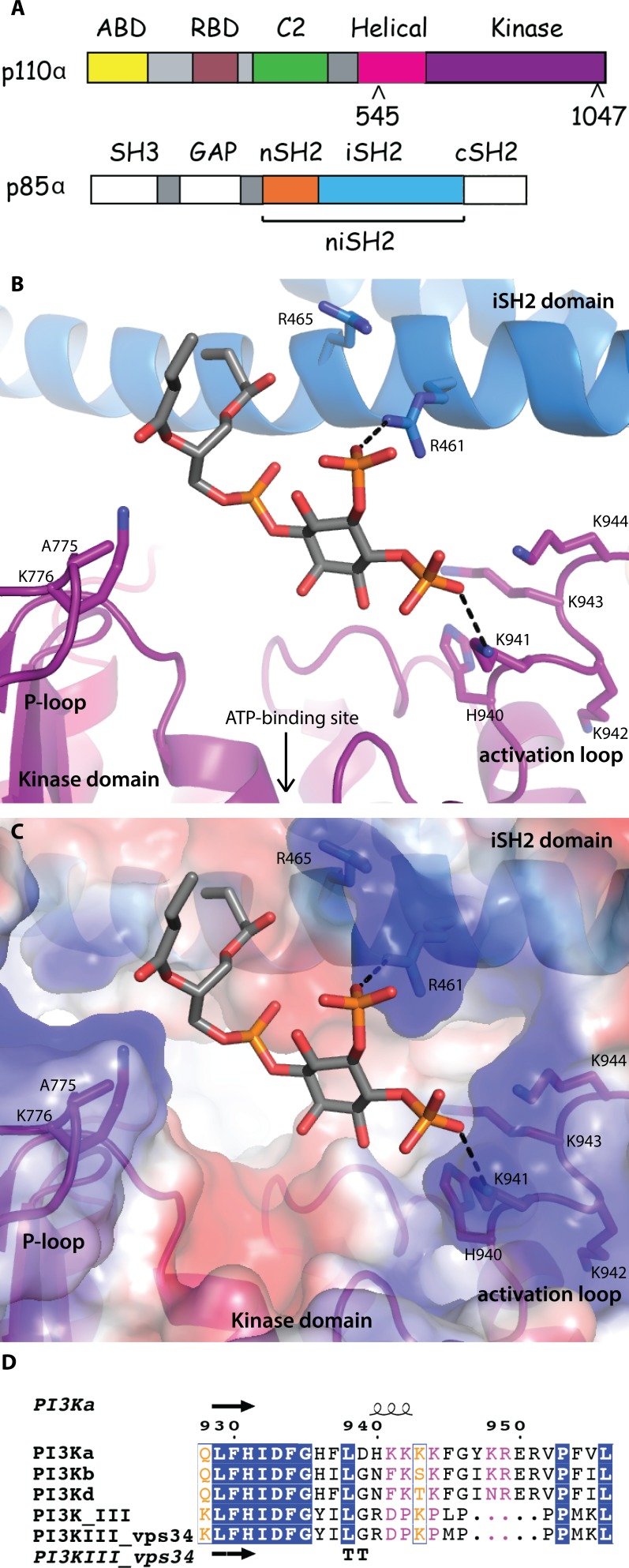
Lipid-binding in PI3K (A) Domain organization of the PI3Kα subunits, p110α and p85α, colored by domain. The positions of p110α hotspot oncogenic mutations are indicated. The niSH2 p85α construct used for crystallography is highlighted. (B) The substrate mimetic diC4-PIP_2_ binding site. This site is adjacent to the ATP binding site, between the activation loop and the P-loop of the kinase domain (shown in purple) and the iSH2 domain (shown in blue). (C) PI3Kα surface colored according to electrostatic potential, highlighting the positively charged PIP_2_ binding-site. (D) Activation loop sequence alignment, performed between the Class IA, II and III PI3Ks. Blue represents identical residues, orange represents similar residues, pink represents differences that have been identified as being important for PIP_2_ recognition and binding.

Dysregulation of the phosphatidylinositol 3-kinase (PI3K) pathway plays a critical role in tumor pathogenesis, with up to 50% of human cancers displaying aberrations in signaling [1]. A key modulator of the pathway, PI3Kα, is mutated in a high fraction of breast, colon, brain, head and neck, gastric, and endometrial cancers [2-6]. About 80% of these mutations are somatic, missense mutations concentrated in three hotspots in the p110α catalytic subunit of the protein (Fig. 1A) [7]. Two helical domain mutants, p110αE542K and p110αE545K, become constitutively active through the loss of auto-inhibition by the N-terminal SH2 domain (nSH2) [8, 9]. As such, they are insensitive to further activation by phosphotyrosine peptide binding [8]. In contrast, the kinase domain oncogenic mutant, p110αH1047R, remains sensitive to activation by receptor tyrosine kinases. The mutation enhances membrane binding, resulting in increased substrate accessibility and elevation of the basal lipid kinase activity [8, 10-12].

Since the publication of the first p110γ crystal structures in 1999, more than 80 crystal structures of the four Class I PI3K isoforms (PI3Kα, PI3Kβ, PI3Kγ and PI3Kδ) have been deposited in the Protein Data Bank (PDB) [13]. The structures of PI3K in complex with ATP and with a wide range of pan-PI3K and isoform selective inhibitors, spanning multiple chemical classes, have been determined [14]. Despite this large amount of structural information, the lipid substrate binding site has yet to be identified crystallographically. Biochemical data has established an important role for the positively charged residues of the activation loop in determining substrate specificity and lipid kinase activity [15, 16]. Replacement of the activation loop in PI3Kα with that of Class II or III PI3K renders PI3Kα unable to phosphorylate PIP_2_. Lipid binding appears to be unaffected, however, as pre-incubation of these hybrids with PIP_2_ prevented binding of the covalent inhibitor, wortmannin [15]. Modeling studies of p110α and p110γ support the biochemical data, placing the phospholipid head group between the positively charged residues of the P-loop equivalent of PI3K (hereafter P-loop; p110α residues 772-778) and the activation loop (p110α residues 935-958), with the 3’-hydroxyl group positioned for phosphate transfer near the γ-phosphate of ATP [13]. Computational studies have postulated a role for p110α K776 within the P-loop in determining phosphoinositide substrate specificity [15]. Class II and III PI3Ks, which cannot phosphorylate phosphatidylinositol-4-5-bisphosphate (PIP_2_), do not have an analogous positively charged residue at this position.

The most common heterodimer construct used for PI3Kα structure determination is full length p110α with a truncated p85α consisting of the nSH2 and the iSH2 domains (p85α residues 322-600), hereafter referred to as p110α/niSH2 (Fig. 1A) [11, 17]. The first insights into the mechanism of nSH2 domain-mediated auto-inhibition were gained from the structures of the p110 oncogenic mutant, H1047R [10]. These structures revealed that phosphotyrosine peptides bind at the interface of the helical and nSH2 domains, competing with the interaction of the nSH2 with the kinase domain and leading to release of its inhibition [10, 18]. However, precisely what effect this has on the conformation of the kinase domain, if any, and how this release results in activation is still not known. Recently, two groups have reported structures that include density for the nSH2 domain, the first within a p110α-p85niSH2 fusion construct (PDB IDs 4L1B, 4L23, 4L2Y), and the other within a double Ras-binding domain (RBD) mutant (M232K, L233K) (PDB ID 4JPS)[19, 20]. However, the structure of the nSH2 domain in complex with wild-type p110α has not yet been determined.

Herein we report the structure of wild-type p110α/niSH2, free and in complex with the truncated lipid substrate mimetic, di-C_4_-phosphatidylinositol-4,5-bisphosphate (diC4-PIP_2_), refined to 2.9 and 3.4 Å, respectively (Table 1). The structures provide insights into lipid-binding and catalysis by PI3Kα. In addition, differences in the interaction of the nSH2 with the kinase domains between the wild-type enzyme and oncogenic mutant H1047R suggest a possible mechanism for the inactivation of the enzyme by the nSH2 domain and for its release.

**Table 1:** Data collection and refinement statistics

	p110α/niSH2	p110α/niSH2 + diC4-PIP_2_
Data collection		
Space group	p2_1_ 2_1_ 2_1_	p2_1_ 2_1_ 2_1_
Cell dimensions		
a, b, c (Å)	114.7, 116.2, 149.1	114.3, 116.1, 148.7
Resolution (Å)	50.00-2.96(3.01-2.96)	50.00-3.36(3.42-3.36)
R_sym_	0.068 (0.69)	0.103 (0.71)
I / σI	3.6 (3.2)	3.5 (4.4)
Completeness (%)	99.6 (100)	99.9 (100)
Redundancy	7.2 (7.1)	9.0 (9.2)
Unique reflections	41,914	28,636
Total reflections	300,309	256,465
X-ray source		
wavelength	0.9788 Å	0.9788 Å
Refinement		
Resolution (Å)	37.79-2.96	37.45-3.5
No. reflections	39,719	27,082
R_work_/R_free_	0.19/0.27	0.24/0.33
No. atoms		
Protein	10,830	10,584
Ligand	-	83
Water	18	5
B-factors		
Protein	53.82	65.81
Ligand	-	90.00
Water	21.22	78.00
R.m.s. deviations		
Bond lengths (Å)	0.011	0.015
Bond angles (°)	1.7	1.9

## RESULTS

### The lipid-substrate binding site of PI3Kα

The substrate mimetic diC4-PIP_2_ sits in a positively-charged crevice bordering the ATP binding site, located in a groove delimited by the P-loop (p110α residues 772-778) and the activation loop of the kinase domain (p85α residues 457-465) (Fig. 1B, 1C). The 3’ hydroxyl group is oriented towards the ATP-binding site, with no direct interactions with the protein. While the electron density for diC4-PIP_2_ is clear (Fig. S1A), only limited density is present for the side chains of the activation loop residues. The 4-phosphate group extends towards the activation loop, forming either a direct salt-bridge or water-mediated hydrogen bond with K941. One oxygen atom from the 5-phosphate group interacts with the iSH2 domain, forming a salt bridge with R461. The 1-phospho group faces the P-loop and is located 4.8 Å from K776. Although this distance is too long for a direct interaction, the two groups probably form a water-mediated hydrogen bond. Both short hydrophobic tails of the diC4-PIP are positioned in a way that would allow membrane insertion of the endogenous PIP_2_ substrate's long hydrophobic tails. Although neither K776 or K941 are present at the equivalent positions in other Class I isoforms, there are lysine residues at adjacent positions in the other isoforms. This suggests that all Class I isoforms could interact with PIP_2_ in a similar way (Fig. 1D). The binding orientationof diC4-PIP_2_ in the crystal structure is compatible with biochemical data implicating the activation loop in determining substrate specificity [15]. The positively charged residues present in the activation loops of the Class I isoforms are missing in the Class II and III PI3K isoforms. This explains why substrates such as PIP_2_, containing 4- and 5-phosphates, would bind with a much lower affinity to the latter isoforms.

Clear electron density for the activation loop was present in the crystal structure of PI3Kβ (PDB ID 2Y3A) and very recently, in some structures of PI3Kα (PDB IDs 4A55, 4JPS, 4L1B, 4L23, 4L2Y) [11, 19-21]. In the structure of wild-type PI3Kα in complex with PIK-108 (PDB ID 4A55), the conformation of the activation loop was influenced by the inhibitor binding to a non-ATP binding site [11]. The conformations of the activation loop in the other PI3Kα structures, including the two reported here and in ref. 19 and 20, are very similar (Fig. S2A). It is nestled at the interface of the kinase and C2 domains of p110α, with the iSH2 and nSH2 domains of p85α. In protein kinases, activation loop phosphorylation results in activation of the kinase activity of the enzyme [22, 23]. In PI3Ks, however, a similar phosphorylation event has not been shown to occur.

### Catalysis and nSH2 domain inhibition

**Fig 2:**
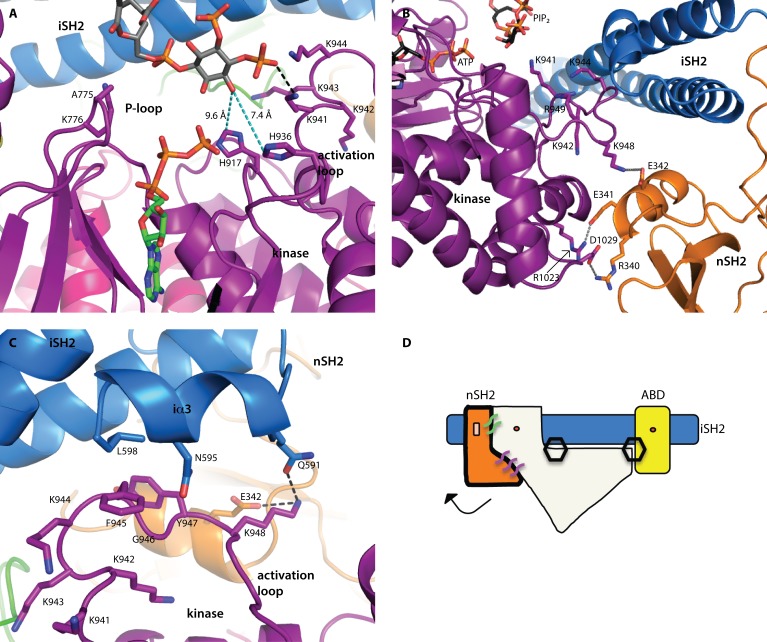
Structural insights into catalysis All domains are colored according to the scheme in Fig. 1A. (A) The relationship between the two substrates was inferred by modeling a molecule of ATP into the binding site (from the alignment with the p110γ-ATP complex structure, PDB ID 1E8X). The 3’-hydroxyl is oriented toward the ATP γ-phosphate. There are two histidine residues in the binding site, which may deprotonate the 3’-hydroxyl for catalysis. Distances are shown in cyan colored dashed lines. (B) The nSH2 domain locks the activation loop in an inactive conformation via a salt-bridge between K948 (p110) and E342 (p85α). (C) The C-terminal residues of the iSH2 (p85α residues 587-602) form a short helix (iα3) which forms an interface with the activation loop. A hydrophobic stacking interaction is made between F945 (p110α) and L598 (p85α). Two key interactions between p85α (E342 and N591) are made with K948 of the activation loop, locking it in an inactive conformation. (D) Schematic representation of the p110α/niSH2 heterodimer showing the general position of the hydrogen bond network that locks p110α in an inactive conformation. In this scheme, p110α is represented in white. The two hexagons represent the PIP_2_ binding sites. The binding of phosphotyrosine residues at the helical-nSH2 interface causes a conformational change that breaks interactions with the activation loop, thereby activating the enzyme. In the basal state, this interface is maintained by key hydrogen bonds or salt-bridges between the subunits, represented by the purple and green lines.

The position of the bound ATP was modeled using a structural alignment of the PIP2 bound structure (PDB ID 4OVV) with the p110γ crystal structure in complex with ATP (PDB ID 1E8X) (Fig. 2A) [13]. In this model, the 3’-hydroxyl group of PIP_2_ is oriented toward the ATP γ-phosphate, in a position ideally suited for phosphate transfer. As in the p110γ-ATP complex structure, D915, the residue analogous to the catalytic base in protein kinases, is too far away to deprotonate the 3’-hydroxyl (10.9 Å), suggesting that in PI3Ks another residue may function as the base [13]. In the PI3Kα structure, there are two histidine residues in proximity to the 3’-hydroxyl, H917 (9.6 Å from the 3’-hydroxyl; equivalent to H948 in p110γ, proposed as an alternative base [13]) and H936 (7.4 Å from the 3’-hydroxyl). Both histidines are conserved in all Class I PI3K isoforms. Neither of these residues are close enough to deprotonate the hydroxyl. H936, however, is located on the activation loop, which is known to be flexible, and may undergo a conformational change that brings H936 into position to deprotonate the 3’-hydroxyl.

Interestingly, a salt-bridge formed between K948 of the activation loop and E342 in the nSH2 domain, suggests a mechanism through which the nSH2 domain inhibits catalytic activity (Fig. 2B): it presumably keeps the activation loop in an inactive conformation. Binding of phosphotyrosine-containing activators at the helical-nSH2 interface dislodges the nSH2 domain from its association with p110, disrupting the contacts with the activation loop and allowing it to adopt an active conformation in closer proximity to the lipid substrate.

The third helix of the iSH2 domain (p85α residues 587-598), iα3, forms an additional interface with the activation loop (Fig. 2C). This interface is mediated by hydrophobic interactions between L598 (p85α) and F945 (p110α), and a hydrogen bond between Q591 (p85α) and K948 (p110α). Deletions (Δ583-602) or truncations (p85-572^STOP^) of this section of the iSH2 domain are known to be oncogenic [24]. Previous studies have suggested that these iSH2 mutations activate the enzyme via a disruption of the iSH2-C2 interface [24, 25]. Hydrogen and deuterium exchange mass spectrometry experiments have suggested that the disruption of this interface is a normal step in the PI3Kα catalytic cycle, and may occur upon membrane binding [12]. These oncogenic deletions appear to function by mimicking this activation step. Our structures (PDB IDs 4OVU, 4OVV) suggest an additional mechanism through which these oncogenic mutations might activate the enzyme: in addition to disrupting the iSH2-C2 interface, the deletion of the iSH2 iα3 helix would release an inhibitory interaction between p85 and the activation loop, weakening the nSH2 domain mediated inhibition of kinase activity (Fig. 2D).

### Interactions of wild-type p110α with the nSH2 domain

Comparison of the structure of the apo wild-type p110α in complex with p85α-niSH2 (reported here) and that of the H1047R mutant reveal key differences in the interactions between p110α and nSH2 [10]. Many of these differences are similar to those between the wild-type and mutant reported by Mandelker *et al.* and will not be discussed further [10]. However, some important differences in the nSH2 and iSH2 domains and their interactions with p110α were not evident in previous studies.

**Fig 3:**
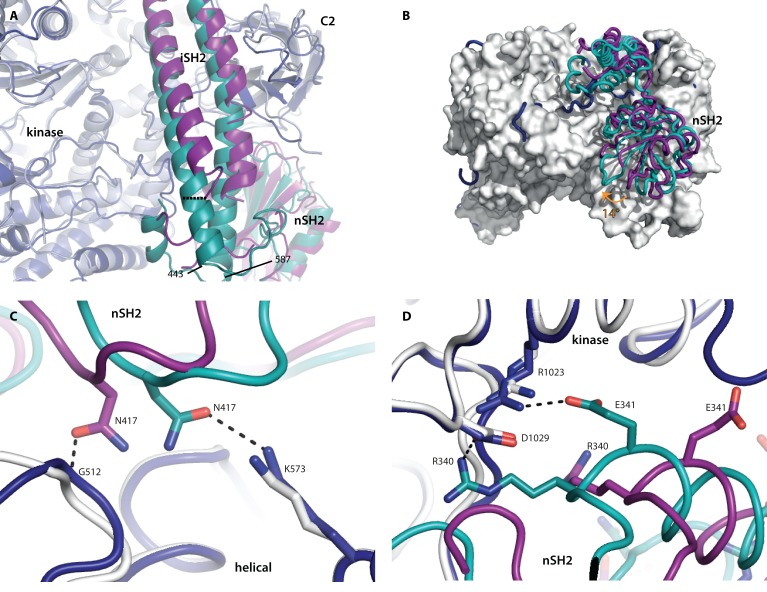
Wild-type p110α has more interactions with the nSH2 domain than the oncogenic mutant H1047R Superposition of the wild-type crystal structure p110α/niSH2 (PDB ID 4OVU) with the p110αH1047R/niSH2 oncogenic mutant (PDB ID 3HHM), obtained by aligning the two p110α molecules. The wild-type p110 and p85α are shown in dark blue and teal, respectively, while the p110αH1047R/niSH2 mutant structure is displayed as light grey (p110α) and purple (p85α). (A) The largest difference between the iSH2 domains is highlighted with a dashed line, measured between the Cα atoms of p85α 450 in each structure. (B) The 14° rotation of the nSH2 domain is identified with an orange arrow. p110αH1047R is shown as a surface representation. (C) Differences in the interactions between the helical and nSH2 domains of the wild-type and oncogenic mutant structures. (D) Two key salt-bridges between the kinase domain and nSH2 are present in the wild-type but absent in the oncogenic mutant structure.

One such difference is a shift of up to 5.5 Å (measured at p85α 450, Fig. S3) in the position of the Cα atoms of the N-terminal helix at the end of the coiled coil iSH2 domain (p85α residues 443-475)(Fig. 3A). The change is less pronounced in the second helix, with an average movement of 2.5 Å (measured at p85α residues 565-579; maximum distance is 3.9 Å at p85α 577, Fig. S3). The positively charged residues on one face of the iSH2 domain, along with two key loops in the kinase domain (p110α residues 723-729 and 863-867) are thought to play a major role in mediating the interaction between PI3Kα and the cell membrane [17]. The iSH2 helices in the oncogenic mutant structure appear to be bent towards the membrane to a greater degree than those in the wild-type, which would be consistent with the increased membrane binding of the oncogenic mutant [10, 11]. These changes may act synergistically with the changes in the loops identified previously to further enhance membrane binding.

Striking differences are also observed in the interaction of the nSH2 domain with p110 (Fig. 3B). In the oncogenic mutant, the nSH2 domain interacts with the C2, helical and kinase domains of p110α, influencing the conformation of these adjacent domains [10, 26]. In the wild-type structure, the nSH2 is rotated 14° towards the kinase and C2 domains. This change results in an increased buried surface area between the nSH2 domain and p110α in the wild-type protein compared to the H1047R mutant (calculated with PISA for the wild-type, 1083 Å^2^, and mutant, 820 Å^2^, considering only the residues present in both structures) [27]. This suggests that the inhibition of the nSH2 domain in the oncogenic mutant may be less potent than in the wild-type protein. The best explanation of these observations can be summarized as follows: the p110 domains differ in their interactions with their nSH2 domains in such a way that the wild-type structure shows extra interactions that result in a tighter contact and consequently greater inhibition (Table S4).

In the helical domain of the wild-type structure, residues p110α K573 and p85α N417 form a hydrogen bond that is not present in the H1047 mutant, because p85α N417 interacts instead with the backbone of p110α G512 (Fig. 3C). A greater difference is observed at the interface between the C2 domain and nSH2, where a number of unique hydrogen bonds are made in each structure. The wild-type nSH2 domain makes three unique hydrogen bonds with the C2 domain, while the oncogenic mutant makes five unique hydrogen bonds with these domains, reflecting the shift in nSH2 conformation (Table S4). Perhaps the most striking difference is the two salt bridges between the kinase domain (R1023 and D1029) and nSH2 (E341 and R340) that are only observed in the wild-type protein (Fig. 3D). These same kinase-nSH2 domain interactions are also present in the higher resolution structures published by Zhao *et al*. [20]. The lack of these key salt-bridges in the oncogenic mutant may reduce the nSH2 domain auto-inhibition in this mutant, resulting in higher lipid kinase activity.

### A second lipid binding site in PI3Kα

**Fig 4:**
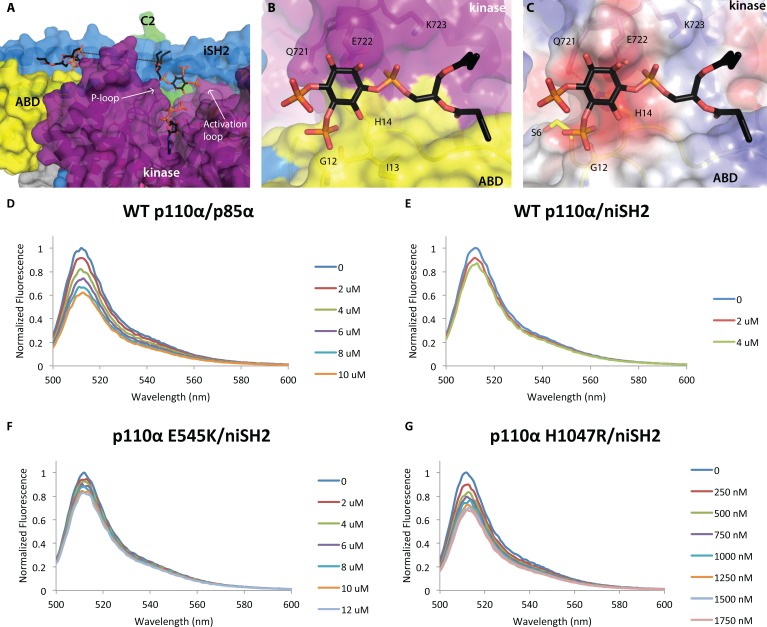
Two PIP2 molecules bound to p110α/niSH2 p110α/niSH2 in complex with diC4-PIP_2_ is shown as a molecular surface with the kinase domain colored in purple, ABD domain in yellow, helical domain in pink, C2 in green, iSH2 in blue and nSH2 in orange. PIP_2_ molecules are shown as sticks with grey carbons. (A) Two molecules of PIP_2_ bind at the interface between p110α and iSH2 of p85α. The distance between the two binding sites is ~21 Å. (B) A second molecule of PIP_2_ binds at the interface between the ABD (yellow) and kinase (purple) domains. (C) The surface of the second PIP_2_ binding site colored according to the electrostatic potential shows a very hydrophobic surface, suggesting possibly a general lipid-binding site rather than a specific PIP_2_ binding site. (Fig. 4D-G) PI3K clusters PIP_2_ in model membrane vesicles containing 50 nM of BODIPY®-FL-PIP_2_. The highest normalized emission intensity corresponds to the vesicles alone. Each subsequent spectrum represents an incremental addition of the corresponding protein. All experiments were performed with N=3. Graphs shown are representative and present the data from a single experiment. (D) Wild-type p110α/p85α quenches 20% of the signal at 4 μM. (E) Wild-type p110α/niSH2 quenches in a similar fashion to the full-length complex. (F) The displayed quenching by p110αE545K/niSH2 is similar to wild-type p110α/niSH2. (G) p110αH1047R/niSH2 quenches the signal with a much higher potency than wild-type. Only 500 nM of protein is required to quench the signal by 20%.

Unexpectedly, electron density for a second diC4-PIP_2_ molecule was observed in the structure of the p110α/niSH2 in complex with the lipid mimetic (Fig. S1B). This second diC4-PIP_2_ molecule binds in a groove between the ABD, kinase and iSH2 domains (Fig. 4A,B). One of the 4’-phosphate oxygens is within hydrogen bonding distance of the backbone of G12 and E722 is 3.6 Å from the 1-phosphate. As with the catalytic PIP2 molecule, the truncated C4-hydrophobic tails are well positioned for membrane binding. The calculated electrostatic potential surface shows very few positive charges, possibly suggesting this may be a general lipid-binding site rather than a specific PIP_2_ binding site (Fig. 4C). Its function may contribute to anchoring PI3K to the cell membrane, but further work is necessary to validate this hypothesis.

To determine whether PI3Kα could indeed bind an additional PIP_2_ molecule within the context of the lipid bilayer of a phospholipid membrane, we measured the ability of PI3Kα to bind and cluster BODIPY® FL-PIP_2_ embedded within phospholipid vesicles. Gambhir *et al.* reported that at a concentration of 0.1% PIP_2_ in 100 nm diameter vesicles, the distance between PIP_2_ molecules is ~300 Å [28]. Self-quenching of the BODIPY® fluorescence occurs when PIP_2_ molecules are brought within 50-60 Å [29]. Therefore if PI3K binds and clusters two or more molecules of BODIPY®-FL-PIP_2_, fluorescence quenching should be observed. A similar approach has been used to demonstrate clustering of BODIPY®-FL-PIP_2_ by MARCKS, NAP-22 and myelin basic protein (MBP) as well as clustering of BODIPY®-TMR-PIP_2_ by dynamin [28, 30-32].

BODIPY®-PIP_2_ fluorescence quenching was measured using unilamellar vesicles with a diameter of 100 nm. The addition of wild-type, full-length PI3K (p110α/p85α) to vesicles containing BODIPY®-FL-PIP_2_ resulted in quenching of the fluorescence signal in a dose dependent manner suggesting PI3Kα can bind and cluster two or more molecules of PIP_2_ (Fig. 4D). Titration of vesicles with p110α/niSH2 (Fig. 4E), a complex devoid of the RhoGap, SH3 and cSH2 domains of p85α, demonstrated fluorescence quenching to the same degree as full length p110α/p85α. There was relatively little quenching by p85α alone in the absence of p110α (Fig. S5). Taken together, these data suggest that there is an additional binding site for PIP_2_ on p110α or at the interface of p110α and p85α subunits of PI3Kα.

We also examined the effects of two oncogenic mutations on the ability of PI3K to bind two PIP_2_ molecules. In similar experiments, p110αE545K/niSH2 showed comparable fluorescence quenching to wild-type p110α/niSH2, suggesting that this mutation does not affect PIP_2_ binding (Fig. 4F). Interestingly, p110αH1047R/niSH2, demonstrated greatly increased quenching compared to wild-type p110α/niSH2 (Fig. 4G). Based on these results, along with previous data and inferences discussed in this work, it seems likely that this effect is due to increased membrane binding of the H1047R mutant.

## DISCUSSION

Binding of phosphotyrosine-containing effectors at the nSH2-helical interface activate PI3Kα catalysis [8, 10]. The structures of wild-type p110α/niSH2 alone and in complex with diC4-PIP_2_ reported here provide insight into how binding of effectors may be communicated to the kinase domain. We have identified key interactions between the nSH2 domain and the activation loop, along with an iSH2-activation loop interface through which the phosphotyrosine binding event may affect the activation state of the kinase domain and increase kinase activity.

In PI3K, lipid substrate specificity is determined by key positive residues on both the activation loop and the iSH2 domain which recognize the 4- and 5-phosphate groups of the lipid substrate, PIP_2_. The importance of these regions has previously been demonstrated biochemically [15, 16]. H936, located on the activation loop, may act as a base to deprotonate the 3’-hydroxyl group as part of the phosphoryl transfer. Binding of a phosphotyrosine peptide at the helical-nSH2 interface would result in the dislocation of the nSH2 domain, releasing the activation loop to close in on the substrate and repositioning H936 to deprotonate the 3’-hydroxyl.

The structure also shows, surprisingly, that PI3Kα binds an additional PIP_2_ molecule. This observation was confirmed by fluorescence quenching experiments. Further studies are required to ascertain the functional relevance of this additional binding site. Regardless, the identification of multiple lipid binding sites provides additional targets that may enable more selective inhibition among the various isoforms, or even between mutants and wild-type PI3Kα.

## METHODS

### Protein expression for crystallization

Sf9 cells were grown in suspension culture in Sf-900 III Serum Free Media (Invitrogen) supplemented with 0.5% penicillin-streptomycin at 27°C. At a density of 4 x 106 cells per milliliter, cells were infected with WT p110α (or p110αH1047R or p110αE545K) and p85α-niSH2 (or p85α) viruses at a multiplicity of infection ratio of 3:2. Media was supplemented with a PI3K inhibitor as described in Mandelker *et al.* [10]. Cells were harvested 72 hours after infection and the cell pellet collected through centrifugation at 900 × *g*. Protein purification was performed as previously described [10, 17].

### p85α Protein expression and purification

Full-length p85α was expressed heterogeneously in *Escherichia* coli as described previously. Briefly, cells transformed with the pGEX 4T plasmid containing an N-terminal glutathione S-transferase (GST) fusion-p85 were grown at 37°C in LB medium. Expression was induced with 1 mM isopropyl β-D-1-thiogalactopyranoside (IPTG). Following 4 hours of induction at 37°C, cells were pelleted and stored at -80°C. Cell pellets were resuspended in PBS, 2 mM DTT, Roche complete EDTA-free protease inhibitor cocktail (Roche Diagnostics GmbH, Mannheim, Germany), pH 7.4 and lysed using a microfluidizer (Microfluidics, Newton, MA). Clarified lysate was incubated with glutathione sepharose HP resin (GE Healthcare) in binding buffer (PBS, 2 mM DTT, pH 7.4), at 4°C for 2 hours with gentle agitation. The GST-tagged p85 was eluted with 10 volumes of elution buffer (50 mM Tris, 150 mM NaCl, 1 mM EDTA, and 10 mM reduced glutathione, pH 8.0). After thrombin cleavage, the p85 was purified by anion exchange Resource-Q anion exchange column (GE Healthcare). p85 was eluted using a linear gradient of 0 – 100% anion exchange buffer B (50 mM Tris, 500 mM NaCl, pH 8.0) over 60 column volumes. p85 of ≥ 90% homogeneity, was loaded onto a HiLoad 26/60 Superdex 200 prep grade gel filtration column (GE Healthcare) equilibrated in gel filtration buffer (50 mM Tris, 300 mM NaCl, pH 8.5); fractions containing p85 of ≥ 95% homogeneity, as determined by SDS-PAGE, were pooled. p85 was concentrated to 15 mg/mL.

### Crystallization and data collection

Crystallization was performed as previously described and improved with successive rounds of macroseeding [17]. Crystals of p110α/niSH2 were soaked for one hour with 1 mM diC4-phosphatidylinositol-4,5-bisphosphate (Echelon Biosciences). X-ray diffraction data were collected at beamlines X6A and X25 of NSLS at Brookhaven National Laboratory. The crystals diffracted to a resolution of 2.96 Å in the absence of diC4-PIP_2_ and 3.37 Å in the presence of diC4-PIP_2_. Data were processed with HKL2000 (Table 1)[33].

### Structure determination and analysis

The free structure was determined by using the coordinates of the previously determined WT p110α/niSH2 (PDB ID 2RD0) [17] as a model. After rigid body and positional refinement, the program Coot [34] was used for model building. Initial refinement revealed that the nSH2 domain of p85α was present and ordered in the structure. Therefore, the nSH2 domain of p110αH1047R/niSH2 structure (PDB ID 3HHM) [10] and the p85α nSH2 crystal structure (PDB ID 2IUG) [35] were used as a guide to fit the additional electron density in this region. Iterative rounds of refinement using REFMAC 5.0 [36-38] yielded a final *R_work_* of 0.191 and an *R_free_* of 0.271 to 2.95 Å in the absence of diC4-PIP_2_. The refined coordinates of the free wild-type structure were used as an initial model for the determination of the structure in the presence of diC4-PIP_2_; refinement yielded an *R_cryst_* of 0.23 and an *R_free_* of 0.33. The overall quality of the final model was assessed by using the programs PROCHECK [39] and WHATIF [40]. Visualization, analysis and figure preparation were carried out with PyMOL (The PyMOL Molecular Graphics System, Version 1.5.0.1 Schrödinger, LLC). Sequence alignments were done using Clustal Omega and colored with EsPRIPT [41]. The calculation of the buried surface was done with the program PISA [27].

### Preparation of BODIPY-FL-PIP_2_ vesicles

Unilamellar vesicles containing PC/PE/PS/BODIPY-FL-PIP_2_/cholesterol with a molar ratio of 54.9/25/5/0.1/15 were generated as follows: Lipids were dried under a stream of nitrogen in a glass test tube then held under vacuum for 24 hours to completely remove organic solvents. Lipids were then heated in the presence of assay buffer (50 mM Tris pH 7.6, 150 mM NaCl, 1 mM EDTA, 2 mM DTT) at 50°C for 10 mins. Lipids were resuspended by vortexing then extruded through a polycarbonate filter with 100 nm pore sizes to yield unilamellar vesicles 100 nm in diameter. BODIPY®-FL-PIP_2_ was purchased from Echelon Biosciences. All other lipids were purchased from Avanti Polar Lipids.

### Fluorescence quenching experiments

BODIPY®-FL-PIP_2_ containing vesicles were diluted to a final concentration of 50 μM lipid (50 nM BODIPY®-FL-PIP_2_) in assay buffer (50 mM Tris pH 7.6, 150 mM NaCl, 1 mM EDTA, 2 mM DTT) for all assays. Ovalbumin (0.5 mg/mL) was added to the assay solution to minimize signal loss due to vesicle binding to glass surfaces during mixing and binding measurements. Fluorescence measurements were taken on a FluoroLog fluorometer from HORIBA scientific. BODIPY®-FL-PIP_2_ was excited at 490 nm and emission readings were recorded from 500 to 600 nm. The spectral bandwidths for excitation and emission were 2 and 5 nm respectively. All binding experiments took place at 25°C in a quartz cuvette. Quenching signals were normalized to the fluorescence signal from the vesicle solution alone and adjusted for dilution due to the addition of the corresponding protein.

## SUPPLEMENTARY MATERIALS FIGURES AND TABLES


