# Supplementary material for: Structural basis of nSH2 regulation and lipid binding in PI3Kα
Source: Oncotarget. 2014 Jul 25;5(14):5198–208. doi: 10.18632/oncotarget.2263 (PMC4170646; doi:10.18632/oncotarget.2263)
Supplement: Supplementary file 1 [file oncotarget-05-5198-s001.pdf]

Structural basis of nSH2 regulation and lipid binding in PI3Kα

Supplementary Materials

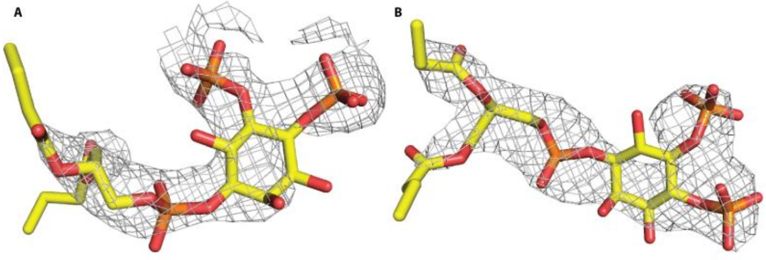

Figure S1: Electron density maps for the two PIP_2 molecules. (A) Electron density map ($2F_O$ - F_C at σ=1) for the substrate PIP_2. (B) Electron density map ($2F_O$ - F_C at σ=1) for the second PIP_2 molecule, which binds at the interface between the ABD, iSH2 and kinase domains.

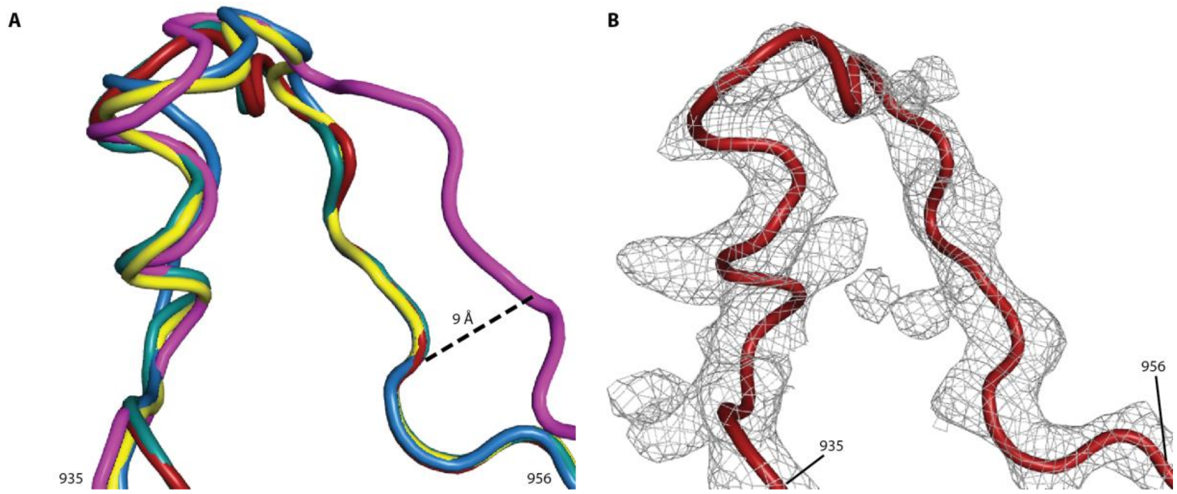

Figure S2: Alignment of activation loops from PI3Kα structures. 4OVU is shown in teal, 4OVV is shown in red, 4JPS is shown in blue, 4L1B is shown in yellow, 4A55 is shown in magenta. All the PI3Kα structures have the activation loop in a similar conformation, with the exception of 4A55, which is influenced by inhibitor binding in the structure.

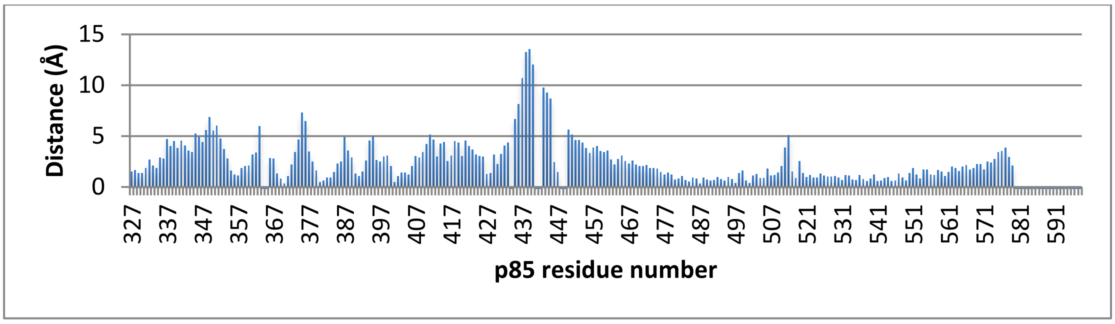

Figure S3: Distance plots. Distance plots of the distance between p85α Cα atoms of wild-type p110α/niSH2 (PDB ID: 4OVU) and H1047R p110α/niSH2 (PDB ID: 3HHM) aligned by Cα atoms of p110α.

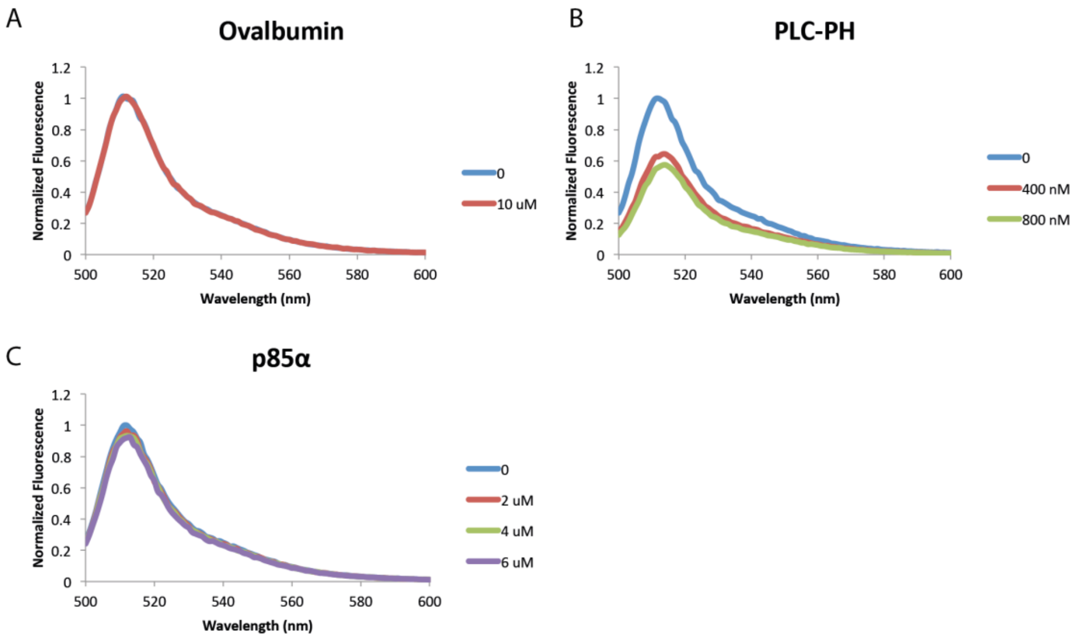

Figure S4: Fluorescence quenching experiments. Model membrane vesicles with 50 nM of BODIPY®-FL-PI(4,5)P_2. The highest emission intensity is normalized to correspond to the vesicles alone. Each subsequent spectrum represents the incremental addition of the corresponding protein. All experiments were performed with N=3. Graphs shown are representative and present the data from a single experiment. (A) Ovalbumin does not quench the fluorescence signal. (B) Positive control of PLCδ (PLC-δ1-PH Domain from Cayman Chemical). (C) p85 showed no quenching of the fluorescence signal at concentrations up to 6 μM.

Table S1: Table of interactions between p110α and nSH2 domain. Residues are colored according to the domains – C2 in green, helical in magenta, kinase in purple.

**p110α residue**	**nSH2 domain residue**	**WT (4OVU)**	**H1047R (3HHM)**
**Arg357**	Asp349		+
**Gly364**	Asn377		+
**Glu365**	Asn377	+	+
**Cys368**	Asp352		+
**Cys368**	Gly375		+
**Glu453**	Arg348	+	+
**Asp454**	Arg348		+
**Asp454**	Asp349	+	
**Asp454**	Arg373	+	
**Leu455**	Asp349	+	
**Glu542**	Arg340	+	+
**Glu545**	Leu380	+	+
**Gln546**	Lys382	+	+
**Gln546**	Phe392	+	+
**Asp549**	Asn417	+	+
**Lys573**	Asn417	+	
**Lys573**	Lys419	+	
**Asn575**	Lys419	+	
**Asp1029**	Arg340	+	
**Arg1023**	Glu341	+	
